# MT2-MMP induces proteolysis and leads to EMT in carcinomas

**DOI:** 10.18632/oncotarget.10194

**Published:** 2016-06-21

**Authors:** Yusi Liu, Xiaojiao Sun, Jinfa Feng, Li-Li Deng, Yihao Liu, Bokang Li, Mingyue Zhu, Changlian Lu, Lingyun Zhou

**Affiliations:** ^1^ Department of Biopharmaceutical Sciences, College of Pharmacy, Harbin Medical University, Harbin, China; ^2^ Department of Pathophysiology, Harbin Medical University, Harbin, China; ^3^ Department of General Surgery, Heilongjiang Province Hospital, Harbin, China; ^4^ Department of Oncology, The Second Affiliated Hospital of Harbin Medical University, Harbin, China; ^5^ Department of Biochemistry and Molecular Biology, Harbin Medical University, Harbin, China

**Keywords:** MT2-MMP, cancer, epithelial-mesenchymal transition (EMT), E-cadherin, zonula occludens-1 (ZO-1)

## Abstract

Epithelial-mesenchymal transition (EMT) is critical for carcinoma invasiveness and metastasis. To investigate the role of membrane-type-2 matrix metalloproteinase (MT2-MMP) in EMT, we generated lentiviral constructs of wild-type (WT) and an inactive Glu260Ala (E260A) mutant MT2-MMP and derived stably transfected HCT116 and A549 cell lines. WT-transfected cells appeared mesenchymal-like, whereas cells transfected with the E260A mutant were epithelial-like, as were cells treated with an MMP inhibitor (GM6001). Expression of E-cadherin, β-catenin, and zonula occludens-1 was lower in cells transfected with WT MT2-MMP compared to vector controls, cells treated with GM6001, or cells transfected with the E260A mutant. An 80-kD N-terminal fragment of E-cadherin was immunoprecipitated in conditioned medium from WT MT2-MMP cells, but not in the medium from vector controls, cells treated with GM6001, or E260A mutant cells. When endogenous expression of MT2-MMP in A2780 human ovarian cancer cells was inhibited using GM6001 or MT2-MMP-specific siRNA, levels of the 80-kD E-cadherin fragment in conditioned medium were decreased. Chick embryo chorioallantoic membrane invasion assays demonstrated that cells transfected with WT MT2-MMP were more invasive than cells transfected with control vector, treated with GM6001, or transfected with the E260A mutant. These results suggest that MT2-MMP degrades adherens and tight junction proteins and results in EMT, making it a potential mediator of EMT in carcinomas.

## INTRODUCTION

Epithelial-mesenchymal transition (EMT) is the transition of immotile, polarized epithelial cells into highly motile, fibroblastoid-like cells. EMT is a critical step for the acquisition of an invasive and metastatic morphology in cancer cells and their ultimate dissemination and colonization in distant locations [1–4]. Epithelial cells are intimately associated with each other through specialized cell-cell contact structures, such as adherens junctions and tight junctions, which are absent in mesenchymal cells [5]. These contacts are disrupted during the process of cancer EMT.

E-cadherin is a transmembrane glycoprotein that homodimerizes on the cell surface in adherens junctions. E-cadherin homodimers interact with other homodimers on an adjacent cell via extracellular domains to form epithelial cell-cell adhesion [1]. The cytoplasmic domain of E-cadherin associates directly with β-catenin and γ-catenin, which link to the actin cytoskeleton through α-catenin [1, 6]. Downregulation of E-cadherin occurs during EMT, and E-cadherin is considered a suppressor of invasion and growth in many epithelial cancers [7, 8]. In addition to transcriptional regulation, E-cadherin is reportedly cleaved by proteases, such as A disintegrin and metalloprotease (ADAM)-10 and -15 [9, 10], plasmin [11], cathepsins B, L, and S [12], kallikreins-6 and -7 [13, 14], and matrix metalloprotease (MMP)-3 and -7 [15, 16].

Tight junctions connecting adjacent epithelial cells near the apical surface act as an intercellular seal that protect against paracellular diffusion. During EMT, tight junctions are also disrupted and tight junction proteins delocalized [17, 18]. Tight junctions are formed by the transmembrane proteins, claudins and occludins, and linked to actin cytoskeletons via ZO-1, -2, and -3 [17, 19, 20]. Truncated mutants of ZO-1 disrupt corneal epithelial cell morphology leading to EMT [21]. Hence, ZO-1 is used as an EMT-related marker [22].

The MMP protein family is comprised of 23 members in humans, and each member has its own substrate, such as collagen, fibronectin, elastin, and laminin. MMPs are involved in embryogenesis, tissue morphogenesis, wound healing, and tumor progression [23–25]. Membrane-Type-2 (MT2)-MMP is membrane-anchored, upregulated in lung adenocarcinoma cells [26] and esophageal cancer [27], and related to cancer progression [28], but the function and substrates of this protease are not known. Herein we demonstrate that MT2-MMP leads to EMT via proteolytic degradation of epithelial cell junctional proteins in carcinomas.

## RESULTS

### Stable expression of MT2-MMP in HCT116 cells results in EMT

**Figure 1:**
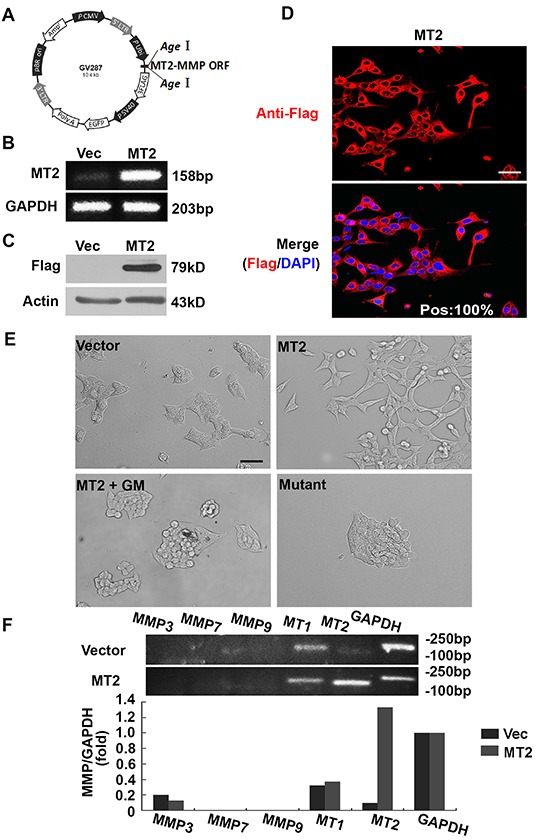
MT2-MMP stable expression in HCT116 cells results in EMT **A.** Lentiviral MT2-MMP vector construction. The open reading frame (ORF) of human MT2-MMP was introduced at the *Age*I/*Age*I site in the GV287 vector containing Ubi-MCS-3FLAG-SV40-EGFP elements to generate stable transfectant cells. **B.** RT-PCR analysis of MT2-MMP mRNA levels in MT2-MMP or vector transfectant HCT116 cell lines. **C.** Western blot analysis of MT2-MMP protein expression in MT2-MMP transfectants, or vector control transfectants, using anti-Flag (1:1000) followed by HRP-conjugated secondary antibody. **D.** Immunofluorescence analysis of MT2-MMP expression (Alexa fluor 594, red; nuclei, DAPI staining, blue). Pos: 100% (the ratio of positive MT2-MMP transfectant cells). Scale bar, 50 μm. **E.** Morphological observations. Stable MT2-MMP transfectants had a fibroblast-like morphology, while vector control transfectants, inactive mutant E260A transfectants, and MT2-MMP transfectants treated with the MMP inhibitor GM6001 (25 μM) all showed typical epithelial morphology. Scale bar, 100 μm. **F.** RT-PCR analysis of MMP3, 7, 9, MT1- and MT2-MMP in HCT116 cell lines transfected with vector or MT2-MMP.

The human MT2-MMP ORF was cloned into lentiviral vector GV287 at the *Age*I/*Age*I site, in order to encode a FLAG epitope-tagged form of human MT2-MMP (Figure 1A). Flow cytometric selection of cells expressing the GV287 vector was performed using GFP. *MT2-MMP* mRNA and protein levels were analyzed using RT-PCR and Western blotting. *MT2-MMP* mRNA levels were increased in MT2-MMP-transfected HCT116 colon cancer cells compared with vector-transfected cells, which had undetectable *MT2-MMP* levels (Figure 1B). The MT2-MMP-flag fusion protein was also expressed at higher levels in MT2-MMP-transfected cells than in the vector-transfected cells (Figure 1C). Almost 100% of MT2-MMP-flag-positive cells were fluorescent (Figure 1D), indicating successful vector construction and selection. Stable cell lines were used for subsequent studies.

HCT116 cells transfected with MT2-MMP adopted an elongated fibroblast-like phenotype, in contrast with cells transfected with empty lentiviral vector, which displayed characteristic epithelial cobblestone morphology (Figure 1E). The effects of MT2-MMP on cell morphology disappeared in the presence of the broad spectrum MMP inhibitor, GM6001 (Figure 1E), suggesting that the effects of MT2-MMP on EMT are dependent upon MMP catalytic activity. Cells transfected with an MT2-MMP inactive mutant (E260A) failed to display a fibroblast-like phenotype (Figure 1E), confirming that the catalytic activity of MT2-MMP was necessary for EMT, regardless of the activity of other proteases, including the ADAM family.

Several MMPs have been reported to induce EMT, including MMP-3 in breast cancer [15, 16], MMP-7 in gastric and lung cancer cell lines [29–31], MMP-9 in ovarian and squamous cancer cell lines [32, 33], and MT1-MMP in an ischemic rat kidney cell line [34]. However MMP-3, -7, and -9 were undetectable, both in cells transfected with vector control and cells transfected with wild-type MT2-MMP (Figure 1F). No significant change in MT1-MMP expression was found. These results suggest that stable MT2-MMP expression resulted in EMT independent of the activity of other MMPs or proteases. Similar results were obtained in an A549 lung cancer cell line (data not shown).

### Overexpression of MT2-MMP results in proteolytic cleavage of E-cadherin in adherens junctions

**Figure 2:**
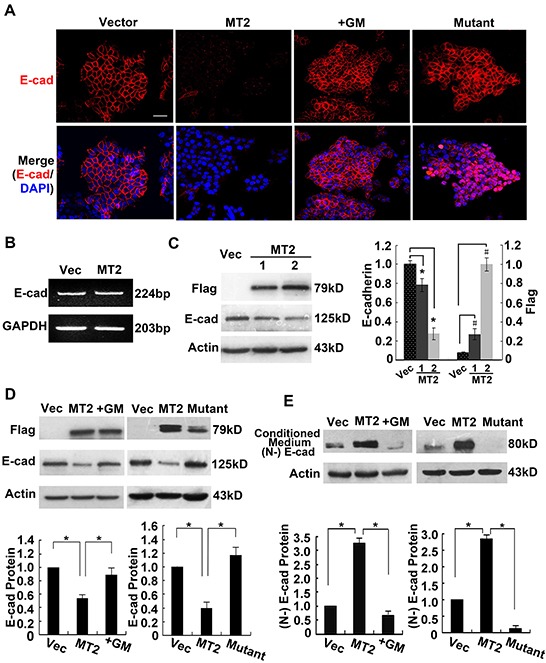
MT2-MMP results in the cleavage and loss of E-cadherin from adherens junctions **A.** Immunofluorescence analysis of E-cadherin protein expression using rabbit anti-E-cadherin antibody (1:50) and Alexa Fluor 594-labeled secondary antibody (1:1000), images captured by confocal laser microscopy (Alexa fluor 594, red; nuclei, DAPI staining, blue). Scale bar, 50 μm. **B.** RT-PCR analysis of E-cadherin mRNA levels in MT2-MMP transfectants versus vector transfectants, showing no changes between them. **C.** Western blot analysis of E-cadherin protein expression in MT2-MMP transfectants (1, without selection by flow cytometry; 2, selection by flow cytometry) or vector control transfectants, using a primary antibody rabbit anti-E-cadherin (1:1000) followed by HRP-conjugated secondary antibody. **D.** Western blot analysis of E-cadherin levels in vector cells, MT2-MMP cells, inactive mutant E260A cells, and GM6001 (25 μM) treated MT2-MMP cells. **E.** Immunoprecipitation and Western blot analyses of the ectodomain of E-cadherin in the conditioned medium of indicated transfectants. Goat anti-E-cadherin antibody (N-20, combination with N-terminal extracellular domain of E-cadherin; Santa Cruz) was used in both immunoprecipitation and Western blot assays. Mean ± SEM, n = 3. * and ^#^ represent *P* < 0.05.

E-cadherin is the most important marker for epithelial cells, so we asked whether MT2-MMP downregulates E-cadherin expression using immunofluorescence staining for E-cadherin. Figure 2A shows that cells transfected with control vector had clear E-cadherin staining between cell boundaries, while this staining was not observed in MT2-MMP-transfected cells. The inhibitor, GM6001, blocked the loss of E-cadherin in MT2-MMP-transfected cells (Figure 2A), indicating that MT2-MMP catalytic activity is necessary for E-cadherin loss. Loss of E-cadherin was also not observed in cells transfected with E260A mutant MT2-MMP (Figure 2A), further confirming that MT2-MMP downregulates E-cadherin expression. Similar results were obtained in A549 cell lines (Supplementary Figure S1).

RT-PCR and Western blotting were performed to detect E-cadherin mRNA and protein levels and to determine whether MT2-MMP inhibited E-cadherin expression on a transcriptional or post-transcriptional level. E-cadherin mRNA expression was not altered (Figure 2B), while E-cadherin protein was decreased, in cells transfected with MT2-MMP as compared with vector controls (Figure 2C). Compared to the MT2-MMP stably transfected cells, cells without selection by flow cytometry had lower MT2-MMP levels, accompanied by higher E-cadherin levels (Figure 2C). Thus, MT2-MMP expression and activity correlates with E-cadherin levels. E-cadherin levels in cells treated with GM6001 or transfected with the E260A inactive mutation were comparable to vector control cells, indicating that active MT2-MMP is necessary for a reduction in E-cadherin (Figure 2D).

E-cadherin is cleaved in the extracellular domain by several MMPs and an 80-kD fragment is released into supernatant. Therefore, E-cadherin cleavage products were immunoprecipitated from conditioned media using an antibody to the extracellular (N-terminal) domain and analyzed using Western blotting. An 80-kD N-terminal fragment of E-cadherin was detected in WT MT2-MMP-transfected cells, but not in the presence of the inhibitor, GM6001, or in cells transfected with either the control vector or the E260A mutant (Figure 2E). These results indicate that active MT2-MMP cleaves E-cadherin protein, resulting in the release of the N-terminal fragment into culture medium and the loss of E-cadherin from adherens junctions.

### Suppression of endogenous MT2-MMP inhibits cleavage of E-cadherin

**Figure 3:**
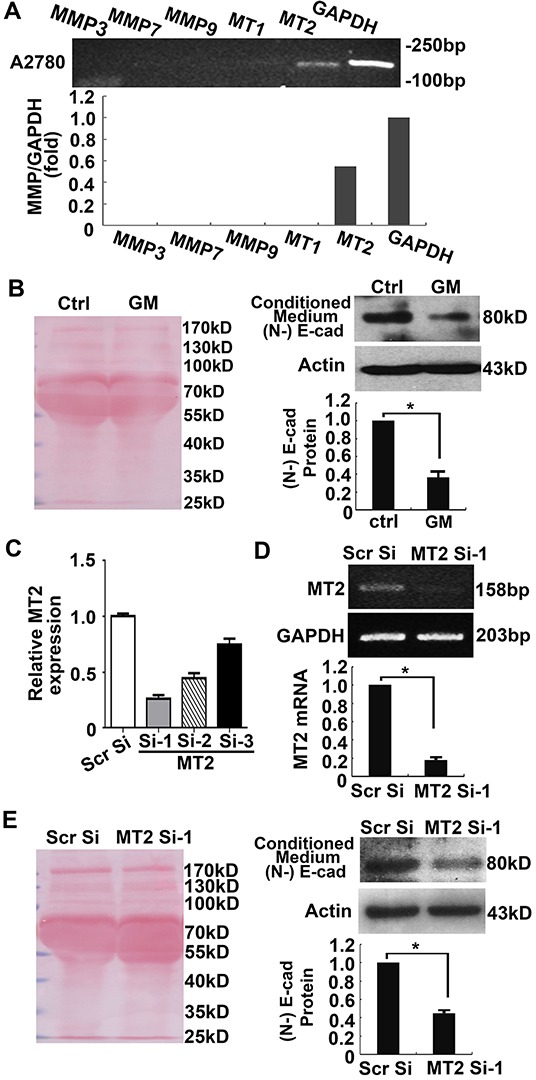
Suppression of endogenous MT2-MMP inhibits E-cadherin shedding in A2780 cancer cells **A.** RT-PCR analysis of MMP3, 7, 9, MT1- and MT2-MMP in the A2780 cancer cell line. **B.** Shed E-cadherin N-terminal fragments in the conditioned medium were examined by Immunoprecipitation and Western blotting using anti-N-terminal E-cadherin antibodies (right panel). Cells were lysed and actin was detected as a loading control (right panel). Ponceau staining of the membrane with samples from the conditioned medium was used as a loading control (left panel). **C, D.** The effect of 3 pairs of siRNAs against MT2-MMP was analyzed by Real-time PCR (C). The best inhibition was achieved by Si-1 (D). **E.** Shed E-cadherin in the conditioned medium was examined following knockdown of MT2-MMP using MT2 Si-1. Left panel, Ponceau staining. Right panel, Western blotting with anti-N-terminal E-cadherin antibody. Cells were lysed and actin was detected as loading control. Mean ± SEM, n = 3. *P < 0.05.

Next we investigated whether endogenous MT2-MMP has the ability to induce cleavage of E-cadherin. In the A2780 ovarian cancer cell line, MT2-MMP expression was detectable by RT-PCR, but there was no expression of other MMPs, including MMP-3, -7, -9 and MT1-MMP, which reportedly correlate with EMT (Figure 3A). A2780 cells were treated for 2 days with the MMP inhibitor, GM6001, conditioned medium was collected, and E-cadherin breakdown products were analyzed by Immunoprecipitation and Western blotting using an N-terminal E-cadherin antibody. Ponceau staining of membranes (Figure 3B, left panel), and actin expression as detected by Western blotting (Figure 3B, right panel) were used as loading controls. As shown in Figure 3B, the 80-kD N-terminal fragment of E-cadherin was reduced in the GM6001 treated conditioned medium when compared to the control medium. Similar results were obtained using the SKOV3 ovarian cancer cell line (Supplementary Figure S2).

To further confirm MT2-MMP proteolytic activity on E-cadherin, specific small interfering RNAs (siRNAs) against MT2-MMP were transfected into A2780 cancer cells. Among the 3 different siRNAs, MT2 Si-1 presented the strongest inhibitory effect on MT2-MMP expression (Figure 3C, 3D). As expected, transfection of MT2 Si-1 into A2780 cancer cells, inhibited E-cadherin shedding into the culture medium when compared with scramble siRNA (Figure 3E), suggesting that endogenous MT2-MMP has the ability to cleave E-cadherin.

### MT2-MMP decreases the association of β-catenin with the cell membrane

**Figure 4:**
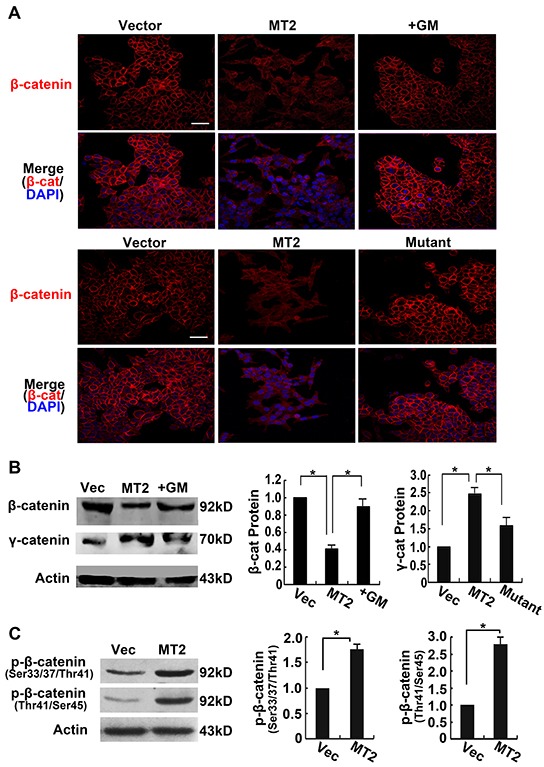
MT2-MMP decreases β-catenin association with the cell membrane **A.** Immunofluorescence analysis of β-catenin protein expression transfectants using primary antibody rabbit anti-E-cadherin (1:1000) and Alexa Fluor 594-labeled secondary antibody (1:1000), images captured by confocal laser microscopy (Alexa fluor 594, red; nuclei, DAPI staining, blue). Scale bar, 50 μm. **B.** Western blot analysis of β-catenin protein levels using rabbit anti-β-catenin (1:1000) followed by HRP-conjugated secondary antibody. **C.** Western blot analysis of phosphorlated β-catenin (Ser33/37/Thr41 and Thr41/Ser45). Mean ± SEM, n = 3. *P < 0.05.

β-catenin is associated with the cytoplasmic tail of E-cadherin, where it provides structural support in the E-cadherin-catenin-actin skeleton complex and plays a critical role in the maintenance of adherens junctions. Immunofluorescence staining for β-catenin was performed in stably transfected HCT116 cell lines to investigate whether MT2-MMP expression altered β-catenin following E-cadherin cleavage. Figure 4A shows that cells transfected with control vector presented clear and intact β-catenin staining on cellular membranes. This staining was not observed in MT2-MMP transfected HCT116 cells, suggesting that β-catenin is released from E-cadherin-bound sites. In contrast, neither treatment with the MMP inhibitor, GM6001, nor transfection with the E260A mutant inhibited β-catenin localization to adherens junctions (Figure 4A). Similar results were obtained in A549 cell lines (Supplementary Figure S3).

To further detect the outcome of the released β-catenin from adherens junction, β-catenin in the cytoplasm was assayed using Western blotting. Lower levels of β-catenin were observed in MT2-MMP transfected cells compared to the other three treatment conditions (Figure 4B), indicating that the β-catenin did not remain in the cytoplasm. However, γ-catenin released from adherens junction did accumulate in the cytoplasm. Phosphorylation of β-catenin was detected by Western blotting using antibodies specific for phosphorylation at Ser33/37/Thr41 and Thr41/Ser45. As shown in Figure 4C, MT2-MMP enhanced β-catenin phosphorylation at these sites, resulting in β-catenin ubiquitination and degradation.

### MT2-MMP decreases ZO-1 expression in tight junctions

**Figure 5:**
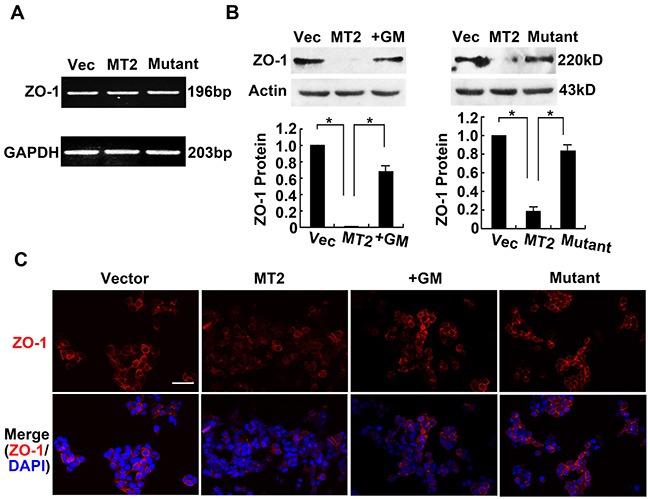
MT2-MMP decreases cellular tight junction ZO-1 expression **A.** RT-PCR analysis of tight junction marker ZO-1 mRNA levels in MT2-MMP transfectants versus vector transfectants, showing no change. **B.** Western blotting analysis of ZO-1 protein levels in vector transfectants, WT MT2-MMP transfectants, inactive mutant E260A transfectants, and MT2-MMP transfectants in the presence of MMP inhibitor GM6001 (25 μM), using primary antibody rabbit anti-ZO-1 (1:1000) followed by HRP-conjugated secondary antibody. Mean ± SEM, n = 3. *P < 0.05. **C.** Immunofluorescence analysis of ZO-1 protein expression using primary antibody rabbit anti-ZO-1 (1:1000) and Alexa Fluor 594-labeled secondary antibody (1:1000), images captured by confocal laser microscopy (Alexa fluor 594, red; nuclei, DAPI staining, blue). Scale bar, 50 μm.

Although MT2-MMP cleaves E-cadherin, resulting in β-catenin loss from the cell surface, we next asked whether MT2-MMP also breaks tight junctions on cell borders. ZO-1 is an important tight junction protein, thus the effects of MT2-MMP on ZO-1 expression and localization were detected using RT-PCR, Western blotting, and Immunofluorescence staining. MT2-MMP did not alter ZO-1 mRNA expression (Figure 5A), but did decrease ZO-1 protein levels (Figure 5B). These results indicate that MT2-MMP modifies ZO-1 at the post-translational level. Notably, this effect of MT2-MMP on ZO-1 was not observed in the presence of the inhibitor GM6001 or the inactive MT2-MMP E260A mutant (Figure 5B). Clear immunofluorescence staining of ZO-1 was present on cell borders of HCT116 cells transfected with the control vector, but staining was decreased in the MT2-MMP-transfected cells (Figure 5C). Similar results were obtained in A549 cell lines (Supplementary Figure S4). These results suggest that decreased ZO-1 protein is dependent on the catalytic activity of MT2-MMP.

### MT2-MMP increases tissue invasion *in vivo*

**Figure 6:**
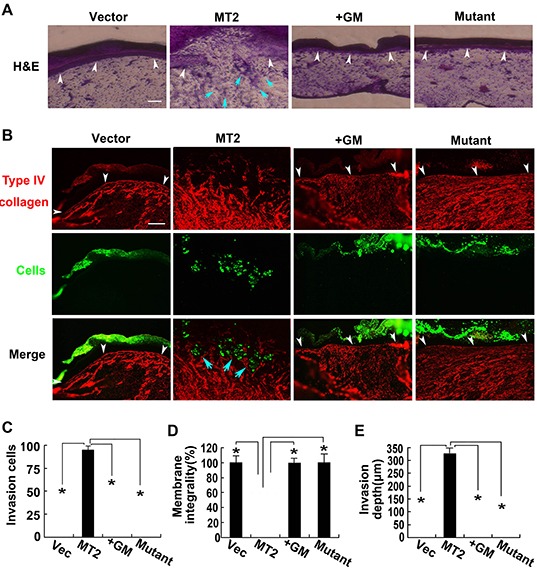
MT2-MMP mobiles HCT116 cell invasion ability *in vivo* **A, B.** MT2-MMP transfectants labeled with green fluoresbrite carboxylate microspheres were placed on the CAM of chick embryos and cultured for 3 d. Samples were stained with H&E staining (A) or anti-type IV collagen antibody and Alexa Fluor 594-labeled secondary antibody (B). White arrowheads mark the CAM upper surface. Blue arrowheads mark the invading cells. Bar, 100 μm. **C.** Number of invasion cells that cross the CAM surface. **D.** Integrality of the CAM upper surface (%). **E.** Invasion depth of leading front of invading cells. mean ± SEM; n=3 *P< 0.05.

To address the possibility that MT2-MMP might play an important role in driving an EMT program capable of promoting an invasive phenotype, HCT116 cells were stably transfected with WT MT2-MMP, E260A mutant, or vector control and fluorescently labeled and cultured atop the chick embryo chorioallantoic membrane (CAM) for 65 hours to detect invasive behavior *in vivo*. Although control vector-transfected HCT116 cells were confined above the CAM upper surface, WT MT2-MMP induced tissue invasiveness (Figure 6A-6E). Treatment of MT2-MMP-transfected cells with the inhibitor GM6001, made cells unable to penetrate the CAM upper surface (Figure 6A-6E). Similarly, cells transfected with E260A mutant were confined above the CAM (Figure 6A-6E). Taken together, our results suggest that MT2-MMP is capable of promoting EMT and cell invasiveness *in vivo*.

## DISCUSSION

Normal multiple layer structures of epithelial tissue and connections such as adherens and tight junctions between cells are obstacles for cell movement. One important prerequisite for the acquisition of invasive and metastatic phenotypes during EMT is the loss or disruption of the connections between cells. A decrease in E-cadherin expression is a typical and critical factor for EMT. Classical E-cadherin decrease arises from several factors, e.g., WNT and transcriptional factors, which repress E-cadherin transcription [35]. However, E-cadherin protein shedding by protease degradation can also induce EMT.

MMPs are comprised of a large family of proteases that play roles in embryogenesis, tissue morphogenesis, wound healing, and cell growth [24, 25]. Among MMP family members, so far only MMP-3, -7 and -9 as well as MT1-MMP correlate with EMT [15, 16, 29–31]. In the present study, MT2-MMP-transfected cells exhibited EMT manifestations and produced an 80-kD N-terminal extracellular fragment of E-cadherin in culture medium, consistent with the soluble E-cadherin produced by MMP-3 and -7 [15, 16]. This 80-kDa ectodomain is detectable in the serum and urine of cancer patients; hence it is a possible biomarker for cancer [36]. In the presence of the MMP-specific inhibitor or E260A mutation, cells failed to produce similar morphological and molecular events to active WT MT2-MMP, suggesting that the EMT is completely dependent on the catalytic activity of MT2-MMP. In addition, no changes were detected in the transcription of either E-cadherin or the fibroblast marker vimentin (data not shown), suggesting that MT2-MMP regulates EMT at the post-translational level.

β-catenin is the linker protein between E-cadherin and actin skeleton, playing a critical role in the maintenance of adherens junctions [37]. In the MT2-MMP-transfected cancer cells, β-catenin was released from E-cadherin, but it did not accumulate in cytoplasm. However neither nucleus translocation nor promotion of proliferation were found in the MT2-MMP-transfected cancer cells (data not shown), suggesting that β-catenin released from adhesion could not participate in Wnt (nuclear) signaling. These findings are consistent with previous studies showing that whether β-catenin participates in adhesion or Wnt signaling is not due to simple competition, but is controlled by distinct molecular forms of β-catenin with different binding properties [38]. In addition, it has been reported in an HCT116 cell line that β-catenin has a mutation at Ser33Tyr (S33Y) [38]. In the present study β-catenin was phosphorylated at Ser37/Thr41/Ser45 as detected by Western blotting, and this phosphorylation may lead to β-catenin ubiquitination and degradation.

ZO-1 is an important molecule for the formation and maintenance of tight junctions [39]. MMP-9 degrades ZO-1 and disrupts tight junctions between endothelial cells [40]. The present study did not find ZO-1 translocation into the nucleus, but Western blotting of whole cell lysates demonstrated that total ZO-1 protein was decreased in cells transfected with MT2-MMP. In contrast, ZO-1 expression changes were not detected in GM6001-treated cells or cells transfected with the inactive mutant MT2-MMP. These results indicate that MT2-MMP degrades ZO-1 directly, which disrupts tight junctions and leads to EMT.

In conclusion, the present study demonstrated that MT2-MMP is a new membrane sheddase that is capable of cleaving extracellular E-cadherin and ZO-1, thereby leading to EMT. Thus, MT2-MMP may be a novel target for cancer therapy. In addition, our findings suggest that the regulation of EMT may occur not only on the transcriptional level, but also on the post-translational level, increasing the number of possible targets of cancer therapy.

## MATERIALS AND METHODS

### Cell culture

The HCT116 human colorectal and A549 lung cancer cell lines were obtained from American Type Culture Collection (ATCC; Manassas, VA, USA). The A2780 human ovarian cancer cell line was obtained from European Collection of Authenticated Cell Cultures (ECACC; London, UK). Cells were routinely maintained in complete medium, Dulbecco's modified Eagle's medium (DMEM; Gibco, USA) supplemented with 10% fetal bovine serum (FBS; Gibco, USA), 2 mM L-glutamine, 100 μ/ml penicillin and 100 μg/ml streptomycin in 5% CO_2_ at 37°C.

### Lentivirus construction

The open reading frame (ORF) of MT2-MMP (Homo sapiens matrix metallopeptidase 15, MMP15; NM_002428.2) was amplified using PCR to generate the MT2-MMP overexpression plasmid vector pGV287-MT2-Flag. The following specific primers were used: forward primer GAG GAT CCC CGG GTA CCG GTC GCC ACC ATG GGC AGC GAC CCG AGCG and reverse primer TCC TTG TAG TCC ATA CCG ACC CAC TCC TGC AGC GAG CG. The underlined nucleotides are required for the ligation, and the bolded nucleotides are complementary to the ORF sequence. Purified PCR products were introduced at the *Age*I/*Age*I site in the GV287 vector, which contains Ubi-MCS-3FLAG-SV40-EGFP elements. Therefore, Western blotting analyzed MT2-MMP protein using a FLAG antibody, and cells were directly visible under fluorescence microscopy. The catalytically inactive MT2-MMP (E/A; Glu260 replaced with Ala) mutant pGV287-MT2-Mut-Flag was generated using specific primers and the QuikChange® II Site-Directed Mutagenesis kit (Stratagene, USA). Constructs were confirmed by sequencing and co-transfected with packaging plasmids into HEK293T cells using Lipofectamine 2000 (Invitrogen, USA). Lentivirus particles in supernatants were harvested and concentrated to a titer of 2 × 10^8^ TU/ml for subsequent infection of HCT116 and A549 cells. The pGV287-Vec lentivirus was generated as a transfection control.

### Transfection and flow cytometric selection

The constructed recombinant lentiviruses, MT2-MMP, E/A mutant, and empty vector control, were transfected into HCT116 and A549 cells using Enhanced Infection Solution supplemented with 5 μg/ml polybrene for 4 h (GeneChem, Shanghai, China). Cells were collected over a 10-d culture period to perform GFP selection using the FACSAria™ II Flow Cytometer (BD Biosciences, San Jose, USA). The stable transfectants HCT116-MT2-MMP, HCT116-E/A mutant, HCT116-vector control, A549-MT2-MMP, A549-E/A mutant, and A549-vector control were obtained. Sorted stable positive cells were used to perform the subsequent expression studies.

### Western blotting and immunoprecipitation

Extracted proteins were separated using 10% SDS-PAGE gel electrophoresis and transferred onto nitrocellulose membranes for 3 h at 200 mA. The membranes were blocked with TBS containing 5% (w/v) nonfat milk and 0.1% (v/v) Tween 20. Membranes were incubated with the following primary antibodies at 4°C: mouse anti-Flag antibody (1:1000; Santa Cruz), rabbit anti-E-cadherin antibody (1:1000; CST, USA), rabbit anti-ZO-1 antibody (1:1000; CST), β-catenin and phosphorlated β-catenin (Ser33/37/Thr41 and Thr41/Ser45) antibodies (1:1000; CST), anti-actin antibody (1:3000; Santa Cruz). Membranes were incubated with the appropriate horseradish peroxidase (HRP)-conjugated secondary antibodies for 1 h at room temperature, and proteins were detected using an enhanced chemiluminescence reagent (ECL; Amersham, IL, USA). Protein band densities were quantified using Quantity One software (Bio-rad, CA, USA) (mean ± SEM, n = 3).

For Immunoprecipitation assays, conditioned medium was collected and centrifuged to deplete cell debris. Supernatants were incubated with a goat anti-E-cadherin antibody (N-20, combination with N-terminal extracellular domain of E-cadherin; Santa Cruz, USA)-treated Protein G agarose beads (Thermo Scientific, MA, USA) at 4°C for 1 h under rotation. Immunoprecipitates were subjected to SDS-PAGE and transferred to membranes followed by Ponceau staining and Western blotting to detect the shed E-cadherin in conditioned medium using N-terminal E-cadherin antibody (N-20). Cells were lysed to perform Western blotting to detect actin as loading control.

### RT-PCR and real-time PCR analyses

Total RNA of cells was extracted using Trizol reagent, and reverse transcribed into cDNA using the Superscript First-Strand Synthesis System (Invitrogen). cDNAs were amplified using standard PCR (Invitrogen) and/or real-time quantitative PCR using SYBR Green Master Mix (Applied Biosystems, USA). The following specific primers were used: (h)MMP3 (forward, 5′-CGGTTCCGCCTGTCTCAAG-3′; reverse, 5′- CGCCAAAAGTGCCTGTCTT-3′; 206-bp product), (h)MMP7 (forward, 5′-GAGTGAGCTACAGT GGGAACA-3′; reverse, 5′-CTATGACGCGGGAGTTT AACAT-3′; 158-bp product), (h)MMP9 (forward, 5′-GGG ACGCAGACATCGTCATC-3′; reverse, 5′-CTCGGCAGAG TCAAAGTGG-3′; 139-bp product), (h)MT1-MMP (forward, 5′-CGAGGTGCCCTATGCCTAC-3′; reverse, 5′-CTCGGCAGAGTCAAAGTGG-3′; 178-bp product), (h)MT2-MMP (forward, 5′-aagagaccaaggagtggatgaa-3′; reverse, 5′-cgtgtagttctggatgctaaagg-3′; 158-bp product), (h)E-cadherin (forward, 5′-CTCACATTTCCCAACTCCTCTC-3′; reverse, 5′-AGCCATCCTGTTTCTCTTTCAA-3′; 224-bp product), (h)ZO-1 (forward, 5′-agccattcccgaaggagttg-3′; reverse, 5′- ccaaccgtcaggagtcatgg-3′; 196-bp product), and GAPDH (forward, 5′-AAGAAGGTGGTGAAGCAGGC-3′; reverse, 5′-TCCACCACCCAGTTGCTGTA-3′; 203-bp product) as a reference. All primers were obtained from Sangon Biotech (Shanghai, China). Real-time PCR was performed in triplicate, and data were analyzed using the method of 2^−ΔΔCT^=(CT_Target_-CT_GAPDH_)_sample_-(CT_Target_-CT_GAPDH_)_control_. PCR products were separated on 2% (w/v) agarose gels. Band densities were analyzed using Bio-Rad Quantity One software (mean ± SEM, n = 3).

### siRNA transfection

siRNAs directed against Homo matrix metallopeptidase 15, MT2 siRNA-1 (NM_002428.3, 1369 - 1388 nt, 5′-CCGACAUCAUGGUACUCUUTT-3′), siRNA-2 (1519 - 1537 nt, 5′-GCACUGACCUGCAU GGAAATT-3′), or siRNA-3 (2213 - 2231 nt, 5′-CCA CACCUUCUUCUUCCAATT-3′) were transfected into A2780 ovarian cancer cells with X-treme GENE siRNA Transfection Reagent according to manufacturer's instructions (Roche, USA), using scrambled siRNA as negative Control (GenePharman, China). MT2-MMP mRNA knockdown efficiency was assayed by Real-time PCR 24 h after transfection with GAPDH as a loading control, and cells and conditioned medium were used for Western blotting and Immunoprecipitation thereafter.

### Immunofluorescence assay

Cells for immunofluorescence assays were cultured in cover glass chambers and rinsed three times in cold phosphate-buffered saline (PBS). Cells were fixed in 4% paraformaldehyde for 5 min and permeabilized with 1% Triton-100 for 3 min. Cells were blocked with 3% goat serum for 45 min at room temperature and incubated with the following primary antibodies overnight at 4°C: rabbit anti-E-cadherin antibody (1:50; CST, USA), rabbit anti-β-catenin antibody (1:1000; CST), rabbit anti-ZO-1 antibody (1:100; CST), and mouse anti-Flag antibody (1:1000; Sigma, USA). Cells were incubated with Alexa Fluor 594-labeled anti-rabbit or anti-mouse secondary antibody (1:1000; Invitrogen, USA) for 1 h at room temperature in a dark room. Nuclei were counterstained using 4′6-diamidino-2-phenylindole (DAPI; 1:10) for 15 min, and fluorescent images were captured using confocal laser microscopy (Olympus, Japan).

### CAM invasion

Cells for *in vivo* cancer cell invasion assays were incubated with green Fluoresbrite carboxylate microspheres (Polysciences, Warrington, PA, USA) and placed on chick embryo chorioallantoic membranes (CAM) for 65 h, as described previously [41]. Frozen sections of samples were assayed with H&E staining and immuostaining. Briefly, immuostaining was performed using an anti-type IV collagen antibody and an Alexa Flour 594-labeled secondary antibody (1:1000; Invitrogen) followed by nucleus counterstaining using DAPI. Fluorescent images were captured using fluorescence microscopy (Nikon, Japan). Cell invasion activities were measured as the number of invasive cells, invasion depth and CAM integrality in three randomly selected fields (mean ± SEM, n = 3).

## SUPPLEMENTARY FIGURES


