# Supplementary material for: MT2-MMP induces proteolysis and leads to EMT in carcinomas
Source: Oncotarget. 2016 Jun 21;7(30):48193–205. doi: 10.18632/oncotarget.10194 (PMC5217011; doi:10.18632/oncotarget.10194)
Supplement: Supplementary file 1 [file oncotarget-07-48193-s001.pdf]

MT2-MMP induces proteolysis and leads to EMT in carcinomas

SUPPLEMENTARY FIGURES

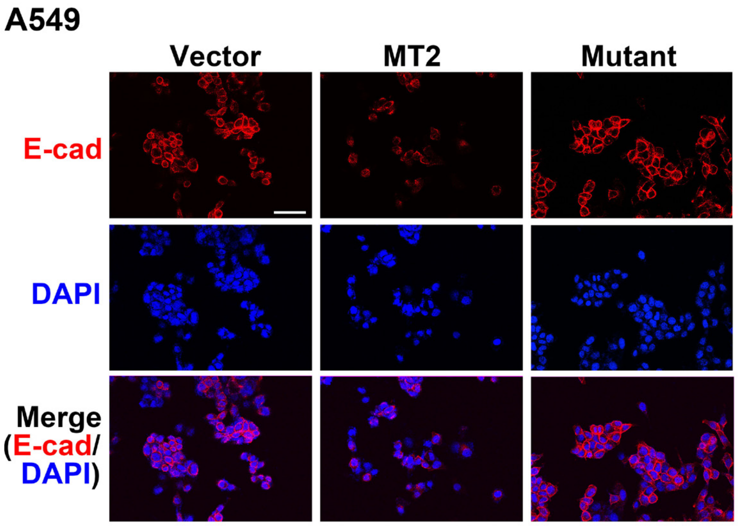

Supplementary Figure S1: MT2-MMP results in the loss of E-cadherin from adherens junctions in A549 cells. Immunofluorescence analysis of E-cadherin protein expression using rabbit anti-E-cadherin antibody (1:50) and Alexa Fluor 594-labeled secondary antibody (1:1000), images captured by confocal laser microscopy (Alexa fluor 594, red; nuclei, DAPI staining, blue). Scale bar, 50 µm.

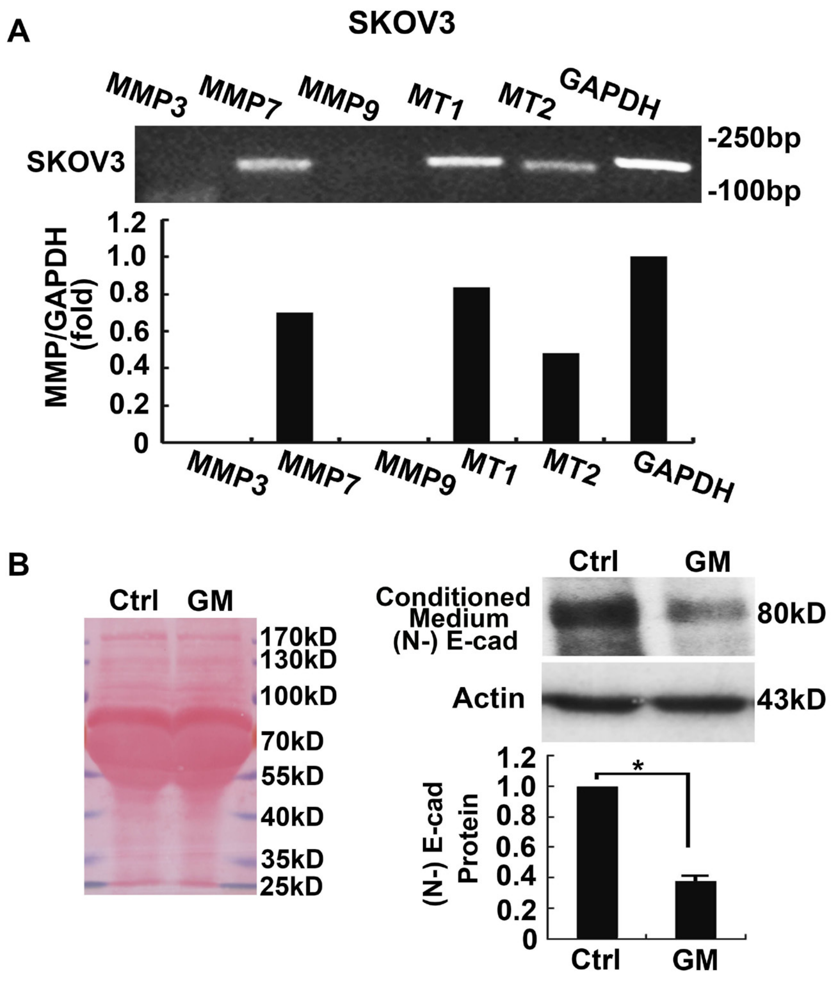

Supplementary Figure S2: Suppression of endogenous MMPs inhibits E-cadherin shedding in an SKOV3 ovarian cancer cell line. A. RT-PCR analysis of MMP3, 7, 9, MT1- and MT2-MMP in an SKOV3 ovarian cancer cell. **B.** E-cadherin N-terminal fragments in the conditioned medium were examined by Immunoprecipitation and Western blotting using anti-N-terminal E-cadherin antibodies (B, right panel). Cells were lysed and actin was detected as a loading control (right panel). Ponceau staining of the membrane with samples from the conditioned medium was employed as a loading control (left panel). mean ± SEM; n=3 *$P < 0.05$.

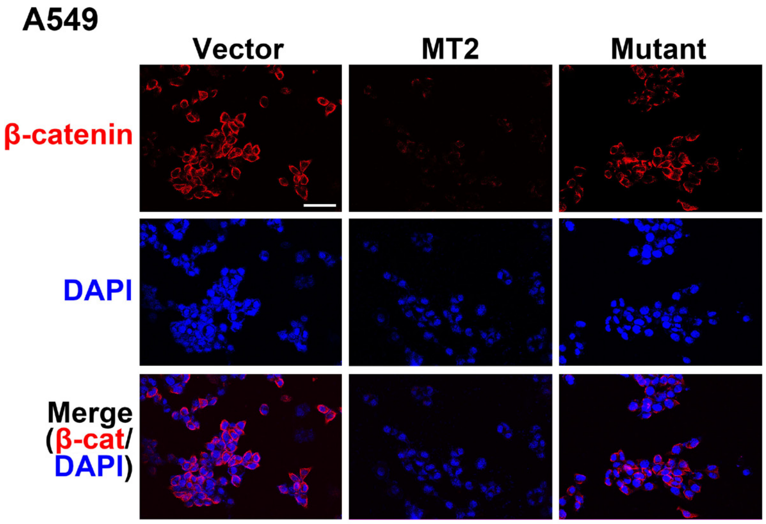

Supplementary Figure S3: MT2-MMP decreases β-catenin association with the cell membrane in A549 cells. Immunofluorescence analysis of β-catenin protein expression transfectants using primary antibody rabbit anti-E-cadherin (1:1000) and Alexa Fluor 594-labeled secondary antibody (1:1000), images captured by confocal laser microscopy (Alexa fluor 594, red; nuclei, DAPI staining, blue). Scale bar, 50 μm.

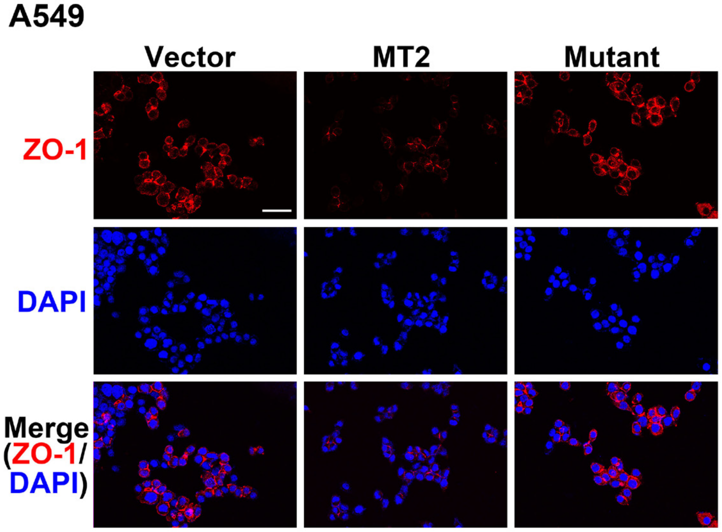

Supplementary Figure S4: MT2-MMP decreases cellular tight junction ZO-1 expression in A549 cells. Immunofluorescence analysis of ZO-1 protein expression using primary antibody rabbit anti-ZO-1 (1:1000) and Alexa Fluor 594-labeled secondary antibody (1:1000), images captured by confocal laser microscopy (Alexa fluor 594, red; nuclei, DAPI staining, blue). Scale bar, 50 μm.
